# Antidepressant-Like Effects of Chronic Guanosine in the Olfactory Bulbectomy Mouse Model

**DOI:** 10.3389/fpsyt.2021.701408

**Published:** 2021-08-04

**Authors:** Roberto Farina Almeida, Yasmine Nonose, Marcelo Ganzella, Samanta Oliveira Loureiro, Andréia Rocha, Daniele Guilhermano Machado, Bruna Bellaver, Fernanda Urruth Fontella, Douglas T. Leffa, Letícia Ferreira Pettenuzzo, Gianina Teribele Venturin, Samuel Greggio, Jaderson Costa da Costa, Eduardo R. Zimmer, Elaine Elisabetsky, Diogo O. Souza

**Affiliations:** ^1^Programa de Pós-Graduação em Ciências Biológicas, Departamento de Ciências Biológicas, Universidade Federal de Ouro Preto, Ouro Preto, Brazil; ^2^Programa de Pós-Graduação em Ciências Biológicas: Bioquímica, Departamento de Bioquímica, Universidade Federal do Rio Grande do Sul, Porto Alegre, Brazil; ^3^Neurobiology Department, Max Planck Institute for Biophysical Chemistry, Göttingen, Germany; ^4^Attention Deficit Hyperactivity Disorder Outpatient Program & Development Psychiatry Program, Hospital de Clínicas de Porto Alegre, Porto Alegre, Brazil; ^5^Preclinical Imaging Center, Brain Institute (Brains) of Rio Grande do Sul, Pontifícia Universidade Católica do Rio Grande do Sul, Porto Alegre, Brazil; ^6^Graduate Program in Biological Sciences: Pharmacology and Therapeutics, Universidade Federal do Rio Grande do Sul, Porto Alegre, Brazil; ^7^Departament of Pharmacology, UFRGS, Porto Alegre, Brazil

**Keywords:** major depressive disorder, psychopharmacology, purines (source: MeSH), guanosine, purinergic signaling, olfactory bulbectomy

## Abstract

Major depressive disorder (MDD) leads to pervasive changes in the health of afflicted patients. Despite advances in the understanding of MDD and its treatment, profound innovation is needed to develop fast-onset antidepressants with higher effectiveness. When acutely administered, the endogenous nucleoside guanosine (GUO) shows fast-onset antidepressant-like effects in several mouse models, including the olfactory bulbectomy (OBX) rodent model. OBX is advocated to possess translational value and be suitable to assess the time course of depressive-like behavior in rodents. This study aimed at investigating the long-term behavioral and neurochemical effects of GUO in a mouse model of depression induced by bilateral bulbectomy (OBX). Mice were submitted to OBX and, after 14 days of recovery, received daily (ip) administration of 7.5 mg/kg GUO or 40 mg/kg imipramine (IMI) for 45 days. GUO and IMI reversed the OBX-induced hyperlocomotion and recognition memory impairment, hippocampal BDNF increase, and redox imbalance (ROS, NO, and GSH levels). GUO also mitigated the OBX-induced hippocampal neuroinflammation (IL-1, IL-6, TNF-α, INF-γ, and IL-10). Brain microPET imaging ([^18^F]FDG) shows that GUO also prevented the OBX-induced increase in hippocampal FDG metabolism. These results provide additional evidence for GUO antidepressant-like effects, associated with beneficial neurochemical outcomes relevant to counteract depression.

## Introduction

Major depressive disorder (MDD) is a multifactorial disorder characterized by a complex symptomatology, leading to important changes in the mental and social health of afflicted patients (1, 2). Despite its high prevalence (3), there are no validated biomarkers that can be used for a differential diagnosis (4). Current antidepressants are characterized by delayed clinical response, significant adverse effects, long-term treatment, and, unfortunately, high relapse rates (3, 5–8). Innovation in the field is sorely needed, especially the development of fast acting drugs with improved effectiveness, for which a better understanding of the pathophysiology underlying MDD is a requirement.

The removal of the olfactory bulbs in rodents induces long-term disruption in pathways of the cortical–hippocampal–amygdala circuit, leading to dysfunctional signaling in limbic areas (9, 10). It recapitulates, in rodents, depressive-like behavioral and neurochemical changes observed in MDD patients (9, 11–13). Adding to its face value, the OBX-induced altered behavior can be reversed or attenuated by chronic (and not acute) treatment with the classical antidepressant agents (12, 14). We proposed (15) that OBX in mice provides two-different windows to explore changes in behavior: an early one (up to 4 weeks after surgery), which includes hyperlocomotion, spatial memory deficits, and anhedonia-like behavior, and a latter one (up to 8 weeks after surgery), where only anhedonia-like behavior is no longer observed. This temporal profile adds to the model's face value by replicating the symptom remission documented in a segment of untreated MDD patients (12, 14).

Depressive patients present decreased levels of serum guanosine [GUO, an endogenous nucleoside with neuroprotective properties (16–18)], corroborating the idea that the purinergic signaling is involved in MDD pathophysiology (18–20). Substantial data have demonstrated that systemic or central GUO induces antidepressant-like effects in distinct rodent models with predictive validity [tail suspension test (21), forced swimming test (21, 22), and acute restrain stress (22)]. GUO antidepressant-like effects were also verified with combined subthreshold doses of GUO and ketamine in the novelty-suppressed feeding test (NSF) (23) and the corticosterone-induced depression models (24). Moreover, a single and acute GUO intraperitoneal administration showed fast-onset antidepressant-like activity, comparable to ketamine, in OBX mice (25). Different mechanisms were postulated for GUO antidepressant effects: interaction with NMDA receptors and the mTOR pathway (21, 25), activation of MAPK/ERK and Nrf2/HO-1 pathways, inhibition of GSK-3β (26), attenuation of oxidative stress (22), and facilitation of neuronal plasticity (27).

As no safe fast-onset antidepressant is currently available for long-term use, a better understanding of long-term administration of GUO is warranted. As the OBX model seems to show higher sensitivity, specificity, and reliability than other experimental depression models (9, 14, 28–30), it was the model chosen for this study.

The purpose of this study is to investigate the effects of chronic GUO in the OBX-induced long-lasting changes in behavior (locomotion and cognition) and brain signaling (glutamate transmission, oxidative stress, and neuroinflammation) parameters. Additionally, positron emission micro-tomography (microPET) with [^18^F]fluorodeoxyglucose ([^18^F]FDG) was used to investigate brain metabolism.

## Materials and Methods

### Animals

Three cohorts of male C57BL/6 mice (45–50 days, 20–25 g) were obtained from Fundação Estadual de Produção e Pesquisa do Rio Grande do Sul, Porto Alegre, Brazil. Animals were housed five per cage and allocated in a room with controlled temperature (22 ± 1°C), under a 12 h/12 h light/dark cycle, and with *ad libitum* access to food and water. The cages were placed in the experimental room 24 h before behavioral tasks for acclimatization. Behavioral tests were carried out between 13:00 and 17:00 h. The [^18^F]FDG-microPET was performed between 7:00 and 11 h in the Preclinical Imaging Center at PUC-RS. All procedures were performed in accordance with the NIH Guide for the Care and Use of Laboratory Animals and approved by the local Ethics Committee (project approval #24577). All efforts were made to minimize suffering and the number of animals used in the experiments.

### Drugs

GUO and imipramine (IMI) were purchased from Sigma Chemicals (St. Louis, MO, USA). All drug solutions were freshly prepared (in saline) before administration and intraperitoneally (i.p.) injected (10 ml/kg), as 7.5 mg/kg (25) for GUO and 40 mg/kg for IMI (used as positive control drug) (31, 32). To reduce the influence of the stress from repeated i.p. administration, the behavioral tests and euthanasia were performed 24 h after the last drug administration. To minimize damages from repeated injections, the abdomen quadrant was changed daily.

### Bilateral Olfactory Bulbectomy

Bilateral olfactory bulb ablation was performed as previously described (15, 25). Briefly, mice were anaesthetized (i.p.) with a combination of xylazine (6 mg/kg) and ketamine (100 mg/kg) diluted in saline. The animals were fixed in a stereotactic frame (Stoelting Co., USA), the skull was shaven, and a burr hole (circa 2 mm in diameter) was made above the olfactory bulbs, 4 mm rostral to the bregma. Both olfactory bulbs were then disconnected with a surgical micro-scissors and removed by suction with a glass Pasteur pipette. Sham-operated mice were treated in the same way, including piercing of the dura mater, but their olfactory bulbs were left intact.

### Treatment Schedule and Behavior

**Figure 1:**
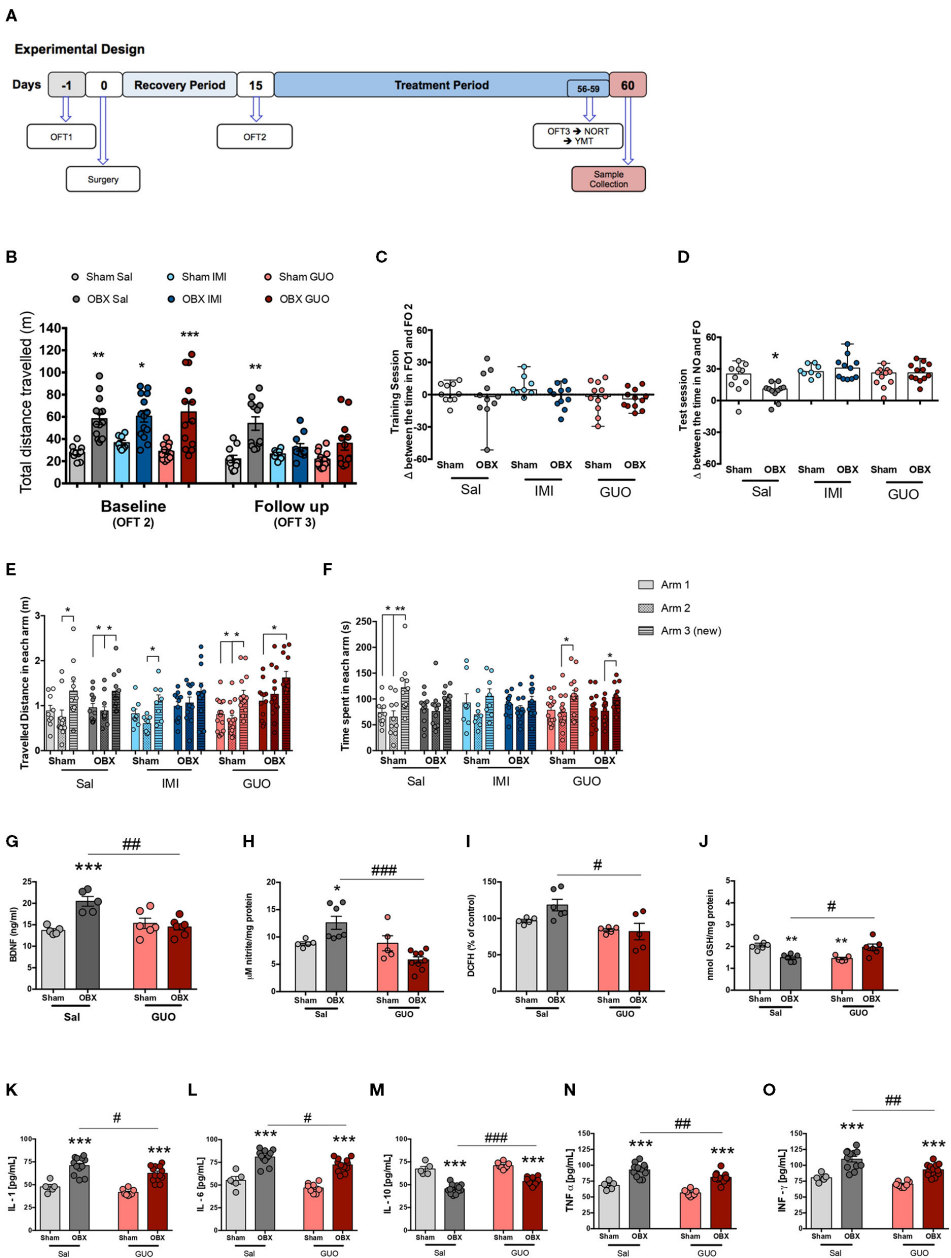
Cohort 1: study design **(A)**. Surgery and treatment effects in locomotor activity in OFT **(B)**: Columns represents mean ± S.E.M. (n = 8–14 mice/group). ^*^p < 0.05, ^**^p < 0.01, ^***^p < 0.001 OBX x respective Sham, two-way ANOVA/Tukey. **(C,D)**: Delta (Δ) between the time spent exploring objects in NORT training (FO1 and FO2) **(C)** and test **(D)** (NO and FO) sessions, respectively. Columns represent median with range (*n* = 8–12). ^*^p < 0.05, ^**^p < 0.01, ^***^p < 0.001, two-way ANOVA/Tukey. **(E,F)**: Total distance travelled **(E)** and time spent in each arm **(F)** in YMT. Columns represent mean ± S.E.M. (*n* = 8–14). ^*^p < 0.05, ^**^p < 0.01, one-way ANOVA/Tukey. Hippocampal levels of BDNF **(G)**, NO **(H)**, DCFH **(I)**, GSH **(J)**, IL-1 **(K)**, IL-6 **(L)**, IL-10 **(M)**, TNF-α **(N)**, **(O)** and INF-γ. Columns represent mean ± S.E.M. (*n* = 5–12). ^*^p < 0.05, ^***^p < 0.001 OBX x respective sham; ^#^*p* < 0.05, ^##^*p* < 0.01, ^###^*p* < 0.001 OBX GUO x Sal; two-way ANOVA/Tukey.

**Figure 2:**
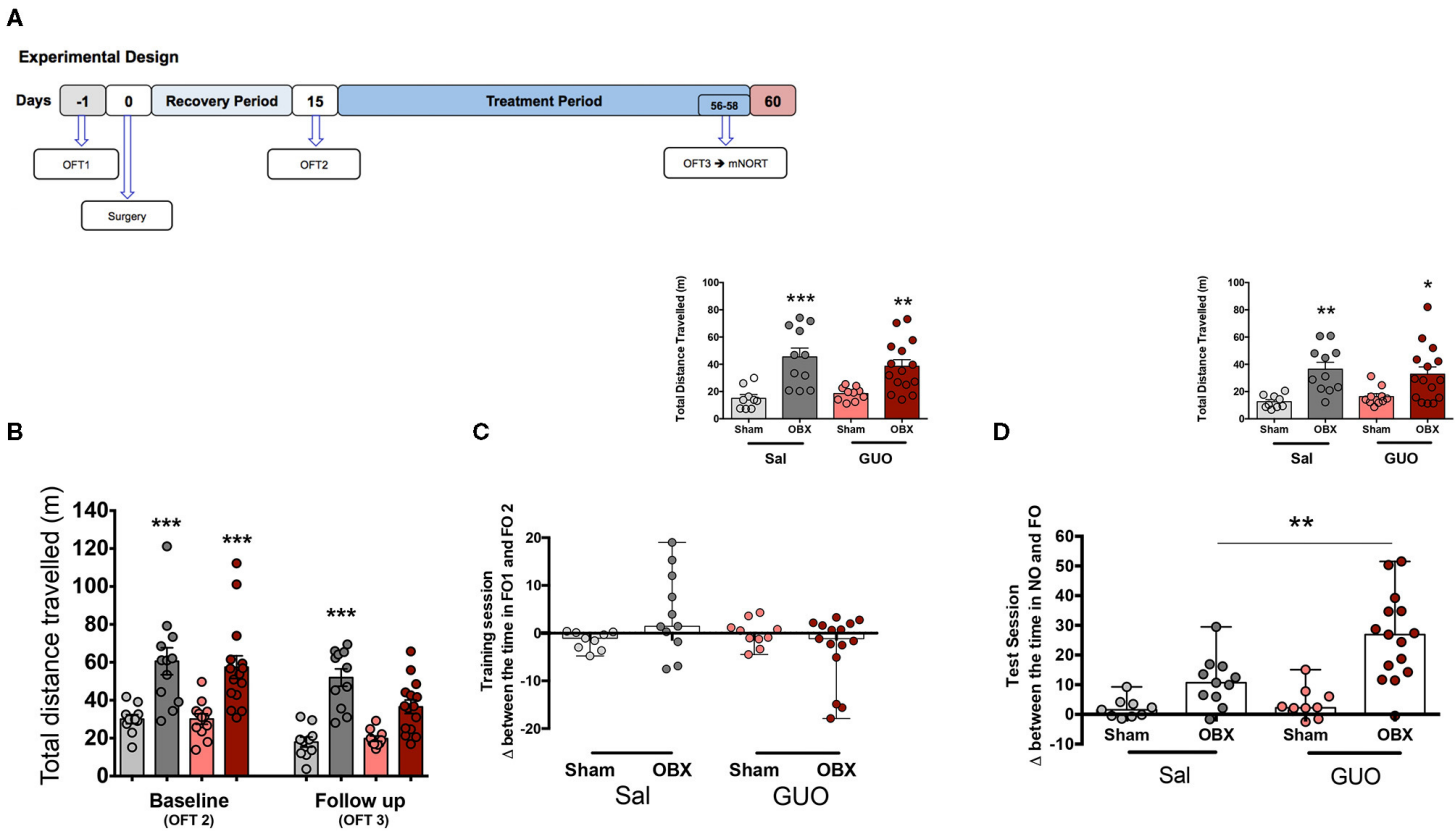
Cohort 2: study design **(A)**. Surgery and GUO treatment effects in locomotor activity in OFT **(B)**. Columns represent mean ± S.E.M. (*n* = 10–15). ^*^p < 0.05, ^***^p < 0.001 OBX x Sham, two-way ANOVA/Tukey. Effects of surgery and GUO treatment at mNORT training **(C)** and test **(D)** sessions. Columns represent median with range, and in the inserts, columns represent mean ± S.E.M. (*n* = 10–15). ^**^p < 0.001; total exploratory behavior, two-way ANOVA/Sidak. Inserts represent distance travelled. ^*^p < 0.05, ^**^p < 0.001, ^***^p < 0.0001 two-way ANOVA/Tukey.

**Figure 3:**
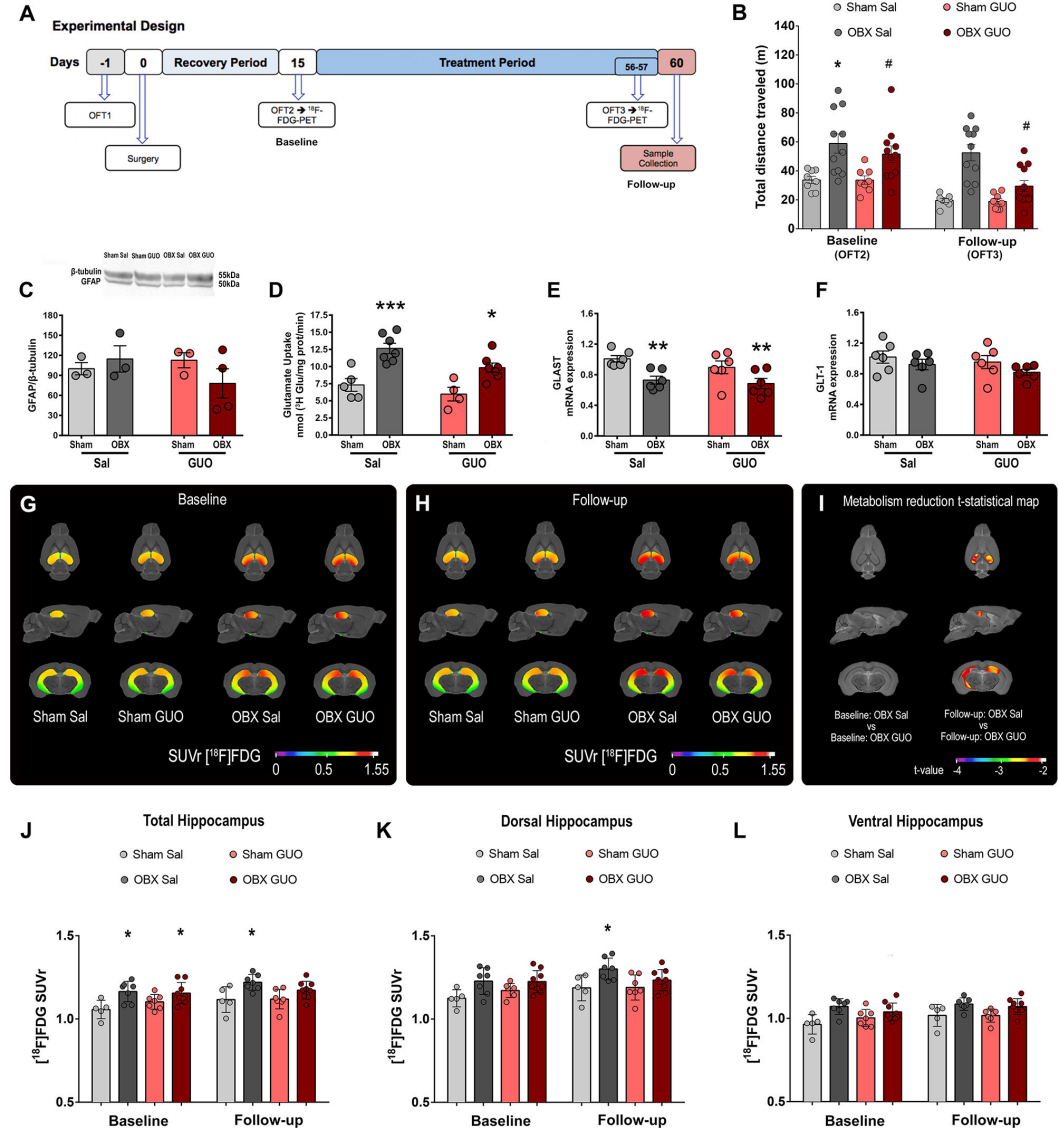
Cohort 3: Study design **(A)**. Effects of surgery and treatment on locomotion at the OFT3 **(B)**. GFAP/β-actin **(C)**, glutamate uptake **(D)**, and mRNA levels of GLAST **(E)** and GLT-1 **(F)** in the hippocampus. Columns represent mean ± S.E.M. (*n* = 8–11 mice/group). *p < 0.05, ^**^p < 0.01, ^***^p < 0.001 Sham x respective OBX; #*p* < 0.05 OBX GUO x Sal, two-way ANOVA/Tukey. Brain metabolic maps showing the mean SUVr in the hippocampus of groups Sham Sal, Sham GUO, OBX Sal, and OBX GUO in the baseline **(G)** and follow-up **(H)** scans. T-statistical maps showing the statistically significant decrease in metabolism in the hippocampal region on the OBX GUO follow-up group in comparison with the group OBX Sal follow-up **(I)**. VOI means [^18^F]FDG SUVr values in the total hippocampal region **(J)**, in the dorsal hippocampal region **(K)**, and in the ventral hippocampal region **(L)**. All images are projected into a standard magnetic resonance imaging (MRI) image in axial, sagittal, and coronal planes. Data represented as mean ± S.D. ^*^p < 0.05 (multiple comparisons—three-way ANOVA with repeated measures).

The research designs can be seen in Figures 1A, 2A, 3A for cohorts 1, 2, and 3, respectively.

In all three cohorts, naïve animals were submitted to the Open Field Task (OFT1) 1 day before surgery. After recovery from surgery (for 14 days), mice were re-submitted to the OFT (OFT2) and a final one (OFT3) after treatments. Daily treatments started immediately after OFT2 and lasted for 45 days, up to 24 h before euthanasia. Behavior or image data collected after the recovery period is considered baseline, and those collected after the treatment are considered follow-up.

From the 80 animals assigned to cohort 1, 12 mice were lost (due to surgical or chronic treatment complications, incomplete bulbectomy, or frontal cortex injury). The following experimental groups were subjected to OFT2 (baseline line): Sham Sal (*n* = 10), OBX Sal (*n* = 14), Sham IMI (*n* = 8), OBX IMI (*n* = 14), Sham GUO (*n* = 14), and OBX GUO (*n* = 13). OFT3 (follow-up) was performed 24 h after daily treatment: Sham Sal (*n* = 10), OBX Sal (*n* = 12), Sham IMI (*n* = 8), OBX IMI (*n* = 11), Sham GUO (*n* = 14), and OBX GUO (*n* = 12). OFT3, the novel object recognition test (NORT), and the Y-maze test (YMT) were performed in these very same groups within 24 h from each other.

From the 50 animals assigned to cohort 2 (*n* = 50), four mice were lost (due to surgical complications, incomplete bulbectomy, or frontal cortex injury). The following groups were subjected to OFT2 (baseline): Sham Sal (*n* = 10), OBX Sal (*n* = 12), Sham GUO (*n* = 12), and OBX GUO (*n* = 15), while OFT3 (follow-up) was performed for Sham Sal (*n* = 10), OBX Sal (*n* = 11), Sham GUO (*n* = 10), and OBX GUO (*n* = 15). The modified NORT (mNORT) was performed 24 h after OFT3.

From the 44 mice assigned to the third cohort, 6 mice were lost (due to surgical complications, incomplete bulbectomy, or for frontal cortex injury). OFT2 (baseline) was performed for Sham Sal (*n* = 8), OBX Sal (*n* = 11), Sham GUO (*n* = 8), and OBX GUO (*n* = 12). OFT3 (follow-up) was performed for Sham Sal (*n* = 8), OBX Sal (*n* = 11), Sham GUO (*n* = 8+), and OBX GUO (*n* = 12). Twenty-four hours after, the OFT3 mice were scanned for [^18^F] FDG-microPET imaging.

#### Locomotor Activity

The OFT was performed as previously detailed (15, 25). Mice were individually placed facing the wall of a gray wooden box (50 × 50 × 50 cm, lighted by 200-lux bulb) and recorded for 10 min by a video-camera (positioned above and at ca. 90° to the square arena) connected to a monitor. The total distance traveled was evaluated using the AnyMaze® software. The apparatus was cleaned with alcohol 70° and dried between trials.

#### Memory

##### Novel Object Recognition Task

The novel object recognition task (NORT) was used to evaluate recognition memory (25, 33). NORT was performed at the same OFT apparatus, consisting of an acquisition trial (training session) and a test trial (test session) performed within a 24 h interval. During the training session (10 min), two identical objects were placed in a symmetric position in the center of the apparatus (subjects with training session exploration time inferior to 20 s were excluded from the experiment). The time spent in each object and the total distance traveled were measured. In the test session (10 min), one of the objects was replaced by a novel object (different shape and material), and again, the time spent exploring each object and distance traveled was measured. Exploration of an object was defined as rearing on or sniffing the object from <1 cm, and/or touching it with the nose. Successful recognition of a previously explored object was reflected by preferential exploration of the novel object in more than 50% of the total time. Experiments were recorded as described in Section **Locomotor Activity**. The total distance in the training and the test sessions were analyzed by AnyMaze® software, while the time spent in each object was analyzed in video records by an experimenter blinded to groups and treatments. After each session, the OFT arena and objects were thoroughly cleaned with 70% ethanol to prevent odor recognition.

##### Modified Novel Object Recognition Task

The purpose of mNORT was to investigate mice ability to discriminate odor, given the potential recovery of olfactory bulbs. The mNORT was conducted as the NORT except for two main differences: i—the objects (FO1 and FO2) at training and test (FO and NO) sessions were kept in rat cages at the animal facility during the 24 h previous to the experiment; and ii—by the exclusion criteria used in NORT, since no Sham mice explored objects at training session more than 20 s.

##### Y-Maze Test

A modified version of the Y-maze test was used (34, 35). The apparatus consisted of three identical arms (30 × 8 cm) disposed at 120°, with gray wooden walls of 15 cm height. The test consisted of a sample phase trial and a test phase trial separated by a 30 min trial interval. In the sample phase trial, each mouse was individually placed in the maze with one of the three arms closed and allowed to explore the other two arms freely for 5 min. At the test trial, each animal was placed again in the maze with all three arms opened and allowed to explore freely for 5 min; the arm closed at sample trial was defined as the new arm. The modified Y-maze was used to evaluate short-term recognition memory. Successful recognition was expressed by increasing the time spent or distance traveled in the new arm when compared to arms 1 and 2. Experiments were video-recorded, and the time spent and total distance traveled in all three arms were analyzed by the AnyMaze® software. After each session, the apparatus was thoroughly cleaned with 70% ethanol.

### Neurochemistry

#### Sample Collection

Twenty-four hours after the last behavioral session and drug administration, mice were anesthetized with xylazine (6 mg/kg) and ketamine (100 mg/kg) and decapitated, brains were removed, and the hippocampi were dissected out for immediate analysis or frozen at −80°C for biochemical evaluations.

#### Redox Homeostase

##### Reactive Oxygen Species

The hippocampus tissue samples were homogenated in phosphate-KCl (20 mM/140 mM) buffer and centrifuged at 1,000 × g × 5 min at 4°C. An aliquot of the supernatant was used to evaluate 2′,7′-dichlorodihydrofluorescein diacetate (DCFH-DA) oxidation (15). DCFH-DA (7 μM) oxidation was determined spectrofluorimetrically. Fluorescence was determined at 488 nm for excitation and 520 nm for emission. A standard curve was carried out using 2′,7′- dichlorofluorescein (DCF). Results are shown as delta of DCFH-DA oxidation between 15 and 30 min of incubation.

##### Nitrite

NO levels were determined by measuring the amount of nitrite (a stable oxidation product of NO) in hippocampal tissue homogenates, as indicated by the Griess reaction. The Griess reagent was a 1:1 mixture of 1% (w/v) sulphanilamide in 2.5% (w/v) phosphoric acid and 0.1% (w/v) N-(1-napththyl) ethylenediamine dihydrochloride in deionized water. Briefly, the tissue was homogenized in phosphate-KCl (20 mM/140 mM) buffer and centrifuged at 1,000 × g × 5 min at 4°C. The supernatant was deproteinized with 20 μl TCA 25%, centrifuged at 2,000 × g × 10 min at 4°C, and immediately neutralized with 2 M potassium bicarbonate. After this procedure, the Griess reagent was added directly to the neutralized sample and incubated in the dark for 15 min at 22°C (15, 36). Samples were analyzed at 550 nm on a microplate spectrophotometer. Nitrite concentrations were calculated using a standard curve, and the results are expressed as percentages relative to the control conditions.

##### Glutathione

GSH levels were assessed as previously described (36). The hippocampal tissues were homogenated in a phosphate-KCl (20 mM/140 mM) buffer containing 5 mM EDTA, and protein was precipitated with 1.7% meta-phosphoric acid. The tissue homogenates were centrifuged at 1,000 × g × 5 min at 4°C, and the supernatants were mixed with o-phthaldialdehyde (at a final concentration of 1 mg/ml methanol) and incubated at 22°C for 15 min. Fluorescence was measured using excitation and emission wavelengths of 350 and 420 nm, respectively. A calibration curve was performed with standard GSH solutions. GSH concentrations were calculated as nmol/mg protein.

#### Neuroinflammation

Hippocampi samples were homogenized in PBS/Tris-HCl/SDS 5% pH 7.4 and centrifuged at 5,000 × g × 10 min at 4°C, and the supernatant was collected (15). Commercial enzyme-linked immunosorbent assay (ELISA) kits for rat IL-1, IL-6, TNF-α, INF-γ, and IL-10 were used according to the manufacturer's instructions (eBIOSCIENCE, San Diego, CA, USA). Briefly, 96-well microplates were incubated with the primary antibody at 4°C overnight, washed, and blocked at room temperature for 1 h. The cytokine standards, calibrators, and samples were added in the plate in triplicate and incubated at room temperature for 2 h. After washing, the secondary antibody conjugated with peroxidase was added and incubated at room temperature for 1 h; the samples were washed and the tetramethylbenzidine chromogen was added. After 15 min, the enzyme reaction was stopped by adding 50 μl phosphoric acid 1 M. The absorbance was measured at 450 nm. The results are expressed as pg/mg protein.

### Glutamate Neurotransmission

#### Glutamate Uptake by Hippocampi Slices

After dissected out, the hippocampi were immediately cut into transverse slices (300 μm thick) using a Mcllwain Tissue Chopper. Transverse hippocampal slices were immediately immersed in HBSS solution (137 NaCl, 0.63 Na_2_HPO_4_, 4.17 NaHCO_3_, 5.36 KCl, 0.44 KH_2_PO_4_, 1.26 CaCl_2_, 0.41 MgSO_4_, 0.49 MgCl_2_, and 5.5 glucose), pH 7.2, 4°C, and glutamate uptake was performed following an adapted protocol (37). Slices were pre-incubated with HBSS, at 37°C for 15 min, followed by medium change and incubation in the presence of 0.2 μCi/ml L-[^3, 4−3^H]glutamate (American Radiolabeled Chemicals, Cat# 0132, Conc. 1 mCi/ml) for 5 min. The incubation was stopped with two ice-cold washes using 1 ml of HBSS, followed by the immediate addition of 200 μl of 0.5 N NaOH, and stored overnight. Na^+^-independent uptake was measured using the same protocol, with modifications in the temperature (4°C) and medium composition (choline chloride instead of sodium chloride). Na^+^-dependent uptake was defined as the difference between both uptakes. The incorporated radioactivity was measured in a Hidex 300 SL scintillation counter. Results are expressed as nMol of glutamate/protein/minute.

#### GLAST and GLT-1 Gene Expression

The gene expression of GLAST and GLT-1 was evaluated in hippocampi from each Sal and GUO groups (*n* = 6) by quantitative real-time polymerase chain reaction (RT-PCR). Total RNA was extracted using TRIzol® reagent (Invitrogen, Carlsbad, CA) following the instructions from the manufacturer. The purity and concentration of the RNA were determined by spectrophotometry at 260/280 nm ratio. One microgram of total RNA was reverse transcribed using the Applied Biosystems™ High-Capacity cDNA Reverse Transcription Kit (Applied Biosystems, Foster City, CA) in a 20-μl reaction. GLAST (Rn01402419_g1), GLT-1 (Rn00691548_m1), and β-actin (Rn00667869_m1) mRNA levels were quantified using the TaqMan real-time RT-PCR system, using inventory primers and probes purchased from Applied Biosystems (Foster City). Quantitative RT-PCR was performed in duplicate using the Applied Biosystems 7500 fast system. No-template and a no-reverse transcriptase were used in each assay as controls, producing no detectable signal during the 40 cycles of amplification. Therefore, target mRNA levels were normalized to β-actin levels using the 2^−ΔΔCt^ method (38).

### Astrocyte Marker

GFAP immunocontent was analyzed by Western blot as previously described (39). Briefly, hippocampal samples from all experimental groups (*n* = 6) were solubilized in ice-cold lysis buffer (4% SDS, 2 mM EDTA, 50 mM Tris-HCl pH 6.8), standardized in sample buffer (62.5 mM Tris-HCl pH 6.8, 2% (*w/v*) SDS, 5% β-mercaptoethanol, 10% (*v/v*) glycerol, 0.002% (*w/v*) bromophenol blue), and boiled at 95°C for 5 min. Samples were separated by SDS-PAGE (35 μg protein/well) and transferred to a nitrocellulose membrane (GE Healthcare). Adequate loading of each sample was confirmed using Ponceau S staining. After blocking with 5% (*w/v*) skim milk overnight, membranes were incubated with primary rabbit antibody one at 4°C (GFAP from Sigma Aldrich, São Paulo/Brazil, 1:3,000 dilution; β-tubulin, 1:2000 dilution), washed, and incubated with horseradish peroxidase-conjugated donkey anti-rabbit IgG (NA934V, 1:5,000 dilution, GE Healthcare, UK) secondary antibody for 2 h. Chemiluminescent bands were detected in ImageQuant LAS4000 system (GE Healthcare) using Immobilon Western chemiluminescence kit (#P90720, Millipore) and quantified with ImageQuant TL software (version 8.1, GE Healthcare). The results are expressed in percentage of control levels after normalization using β-tubulin as an internal standard.

### Brain Derived Neurotrophic Factor

The hippocampi were homogenized in PBS/Tris-HCl/SDS 5% pH 7.4 and centrifuged at 5,000 × g × 10 min at 4°C. BDNF levels were measured in the supernatants by anti-BDNF sandwich-ELISA, in a plate previously coated with anti-BDNF antibody according to the instructions at ELISA Kit for Brain Derived Neurotrophic Factor (Wuhan USCN Business Co., Ltd, Cat. No. SEA011Ra).

### Protein Determination

Protein content was measured using the Pierce BCA® protein kit (Thermo Scientific, Waltham, MA, USA) with bovine serum albumin as standard.

### Glucose Metabolism Imaging Procedure

The PET scans were performed using a Triumph™ microPET at the Brain Institute of Rio Grande do Sul [LabPET-4, TriFoil Imaging, Northridge, CA, USA (for technical information, see Bergeron et al. (40)]. Mice from the third cohort were scanned in two points: after recovery (baseline, 15 days post OBX) and after treatments (follow-up, in experimental day 57) (Figure 3A).

Animals received an intraperitoneal injection of [^18^F]FDG (mean ± s.d. = 25 ± 0.5 mCi) after overnight fasting; each mouse was returned to its home cage for a 40 min period of awake uptake of [^18^F]FDG, immediately followed by a 10 min microPET static acquisition conducted under isoflurane anesthesia (2% at 0.5 L/min oxygen flow). The scan was performed with the animals in a head-first prone position and with the field of view (FOV: 4.6 cm) centered in the animal's head. Throughout these procedures, the animals were kept on a pad heated at 37°C.

Imaging data were reconstructed using the maximum likelihood estimation method (MLEM-3D) algorithm with 20 interactions. Each microPET image was reconstructed with a voxel size of 0.2 × 0.2 × 0.2 mm and spatially normalized into an [^18^F]FDG template using brain normalization in PMOD v3.8 and the Fuse It Tool (PFUSEIT) (PMOD Technologies, Zurich, Switzerland). Further imaging processing and analysis were carried out using the MINC Tool Kit software (www.bic.mni.mcgill.ca/ServicesSoftware). MicroPET images were manually co-registered to a standard mouse MRI histological template. Activity values were normalized by the region of reference showing the lower standard deviation among all the images, the lateral septal nucleus (Supplementary Figure 1A) and, therefore, are expressed as reference standardized uptake value (SUVr). Mean SUVr of total hippocampus and subregions was extracted using predefined VOI templates (Supplementary Figures 1B,C).

### Statistics

Data from OFT, NORT, mNORT, and biochemical parameters were compared by two-way ANOVA followed by Tukey's *post hoc*. Data from YMT was analyzed by one-way ANOVA followed by Tukey's *post hoc*. [^18^F]FDG hippocampal t-statistical maps (voxelwise) were generated comparing groups of interest (*p* < 0.05). Regional values of [^18^F]FDG data were analysed by three way mixed ANOVA (time × surgery × treatment) followed by Newman–Keuls *post hoc*.

## Results

### Cohort 1

Results are shown in Figure 1. All groups of naïve mice, either assigned to sham or OBX surgeries, and any of the treatment groups (saline, IMI, or GUO) presented comparable performances at OFT1 (*F*_(1,74)_ = 0.47 *p* > 0.05) (data not shown). Data from OFT2 (baseline) show that OBX induced hyperlocomotion (*F*_(1,67)_ = 44.99, *p* < 0.0001). For OFT3 (follow-up), the two-way ANOVA identified a main effect of OBX (*F*_(2,61)_ = 4.34, *p* < 0.05), and interaction between treatment and OBX (*F*_(1,61)_ = 23.69, *p* < 0.0001), showing that IMI and GUO reversed the persistent hyperlocomotion of untreated OBX (Figure 1B).

As for NORT memory, two-way ANOVA identified OBX as the main effect (*F*_(5,58)_ = 10.56, *p* < 0.001), and interaction between OBX and treatments (*F*_(2,58)_ = 4.45, *p* < 0.05), showing that IMI and GUO also reversed the OBX-induced NORT memory deficit (Figures 1C,D).

Results in Y-maze task indicate that only OBX IMI group present an impaired performance in distance travelled (*F*_(2,30)_ = 1.49, *p* > 0.05; Figure 1E), while GUO (but not IMI) attenuated the OBX-induced Y-maze memory deficit in time spent in the Y-maze new arm (*F*_(2,33)_ = 3.31, *p* < 0.05; Figure 1F).

Two way ANOVA identified OBX as the main effect for hippocampal BDNF (*F*_(1,19)_ = 10.07, *p* < 0.05; 1G) and nitrite levels (*F*_(1,19)_ = 12.59, *p* < 0.05; Figure 1H); an interaction between OBX and GUO in BDNF, nitrite, and GSH content was identified, with GUO reversing OBX-induced increased in BDNF (*F*_(1,19)_ = 16.50, *p* < 0.05; Figure 1G) and nitrite (*F*_(1,19)_ = 12.76, *p* < 0.05; Figure 1H) levels, and decreased GSH levels (*F*_(1,19)_ = 29.52, *p* < 0.05; Figure 1J). OBX per se did not alter DCFH levels; however, in OBX groups GUO decreased DCFH levels in comparison to saline (Figure 1).

Two way ANOVA shows that OBX clearly induced neuroinflammation at the hippocampus, and GUO slightly attenuated the increase in IL-1 (*F*_(1,33)_ = 66.09, *p* < 0.0001; Figure 1K), IL-6 (*F*_(1,33)_ = 94.15, *p* < 0.0001; Figure 1L), TNF-α (*F*_(1,33)_ = 71.66, *p* < 0.0001; Figure 1N), and INF-γ ((*F*_(1,33)_ = 47.58, *p* < 0.0001; Figure 1O), as well as the decrease in IL-10 (*F*_(1,33)_ = 142.1, *p* < 0.0001; Figure 1M).

### Cohort 2

Replicating the findings from cohort 1 in OFT3 (follow-up), OBX-induced hyperlocomotion (*F*_(1,42)_ = 52.4, *p* < 0.0001; Figure 2B), the interaction between GUO and OBX (*F*_(1,42)_ = 6.06, *p* < 0.05, Figure 2B) shows that GUO reversed the OBX effect on locomotor activity.

Smell is crucial to the performance at NORT. Since olfactory bulbs are known for its neurogenic and proliferative capacity (41–45), and considering that brain circuitry remodeling has been reported after OBX (15), a modified NORT was performed to differentiate the effects of GUO from those of a potential recovery of the sense of smell at follow-up. As expected, at the mNORT, not anosmic sham mice did not reach the exploration criteria, either in training or test session. Corroborating the data from cohort 1, OBX induced memory impairment at NORT test session (*F*_(1,41)_ = 27.73, *p* < 0.0001; Figure 1D), and a positive interaction shows that GUO reversed (*F*_(1,41)_ = 4.97, *p* < 0.05) this deficit. Both not anosmic Sham groups presented decreased locomotion in mNORT training (*F*_(1,41)_ = 27.57, *p* < 0.0001; insert Figure 2C) and test (*F*_(1,41)_ = 19.65, *p* < 0.0001; insert Figure 2D) sessions.

### Cohort 3

Replicating cohorts 1 and 2 findings in OFT3 (follow-up), OBX mice present increases in locomotor activity (*F*_(1,35)_ = 27.76 *p* < 0.0001; Figure 3B) and the interaction between GUO and OBX (*F*_(1,35)_ = 7.360 *p* < 0.05) confirms GUO reversal in OBX-induced hyperlocomotion.

No differences were identified in GFAP protein expression among groups (*F*_(1,19)_ = 2.68, *p* > 0.05, Figure 3C). OBX induced increased hippocampus glutamate uptake (*F*_(1,19)_ = 29.99, *p* < 0.0001; Figure 3D). OBX induced decreased hippocampus GLAST gene expression (*F*_(1,19)_ = 27.57, *p* < 0.0001; Figure 3E), an effect unresponsive to GUO. No differences in GLT-1 gene expression (*F*_(1,19)_ = 0.3115, *p* > 0.05; Figure 3F) were found among conditions or treatments.

At baseline, [^18^F]FDG-microPET showed increased glucose metabolism at the hippocampus of OBX subjects compared to Sham (Hippocampus mean SUVr: Sham Sal = 1.06 ± 0.04, *n* = 5; Sham GUO = 1.08 ± 0.05, *n* = 7; OBX Sal = 1.16 ± 0.05, *n* = 9; OBX GUO = 1.16 ± 0.06, *n* = 11; Figure 3G). The mean hippocampal increase in FDG metabolism driven by OBX was up to 8% in comparison to Sham (hippocampus mean percentage of change: OBX Sal = 8.82%; OBX GUO = 7.93%; Supplementary Figure 1E). A voxel-wise t-statistical analysis, using the Sham Sal group as control, showed that significant ^18^F]FDG-microPET differences were found only for OBX groups (OBX Sal t_(4)_ = 5.76; *p* = 0.006; OBX GUO *t*_(4)_ = 4.25; *p* = 0.013; Sham GUO *t*_(4)_ = 1.21; *p* = 0.292; Supplementary Figure 1F).

At follow-up, [^18^F]FDG-microPET revealed higher hippocampal glucose metabolism for OBX Sal × Sham Sal, while chronic GUO attenuated the OBX-induced rise in [^18^F]FDG signal (OBX Sal × OBX GUO) (hippocampus mean SUVr: Sham Sal = 1.10 ± 0.07, *n* = 6; Sham GUO = 1.11 ± 0.05, *n* = 8; OBX Sal = 1.19 ± 0.05, *n* = 9; OBX GUO = 1.16 ± 0.05, *n* = 10; Figure 3H). At this point, the hippocampal increase in FDG metabolism in the untreated OBX animals reached a peak of 15% increase (mean of 13.8%) in comparison to Sham Sal, while the OBX group treated with GUO presented a mean value of 9% (hippocampus mean percentage of change: OBX Sal = 13.85%; OBX GUO = 9.10%; Supplementary Figure 1G). A voxel-wise t-statistical analysis, using Sham Sal at follow-up as control, only detected a significant increase in FDG metabolism for the saline treated OBX group (OBX Sal *t*_(5)_ = 3.80; *p* = 0.0126); in the group OBX GUO it increased metabolism was only seen in a small cluster (peak *t*_(7)_ = 2.41; *p* = 0.0469) and no significant difference was seen for the group Sham GUO there (peak *t*_(5)_ = 1.65; *p* = 0.1599; Supplementary Figure 1H).

A percentage of change map analysis (data not show) revealed a mean lower hippocampal glucose metabolism for the OBX GUO at follow-up group if compared to the OBX Sal group at the same period (peak *t*_(8)_ of 3.17; *p* = 0.0132; Figure 3I).

A hippocampal mask with 6 VOIs, based on the Allen Mouse Brain Atlas (https://mouse.brain-map.org/), was used to obtain the mean regional [^18^F]FDG SUVr value of the total hippocampus, of the dorsal hippocampus region, and of the ventral hippocampus region. For the total hippocampus mean SUVr (Figure 3J), a three-way ANOVA test—with repeated measures—identified only the effect of OBX [*F*_(1,28)_ = 17.75, *p* = 0.0002], and interaction between OBX and GUO [*F*_(1,20)_ = 5.91, *p* = 0.0245]. The *post hoc* test identified a significant difference for mean total hippocampal SUVr between baseline groups Sham Sal and OBX Sal (*p* < 0.05) and Sham Sal and OBX GUO (*p* < 0.05), and only in Sham Sal and OBX Sal group at follow-up (*p* < 0.05).

At the dorsal region of the hippocampus (Figure 3K), the analysis indicated the effect of the OBX [*F*_(1,28)_ = 12.9, *p* = 0.0012], and interaction between OBX and GUO [*F*_(1,20)_ = 6.4, *p* = 0.0199]. Following, a multiple comparisons test identified a significant difference for mean dorsal hippocampal SUVr only comparing the Sham Sal and OBX Sal groups at follow-up (*p* < 0.05).

Finally, at the ventral region of the hippocampus (Figure 3L), the three-way ANOVA analysis showed an effect for the OBX [*F*_(1,28)_ = 18.32, *p* = 0.0002] but not for treatment [*F*_(1,20)_ = 0.01; *p* = 0.9194] or time [*F*_(1,28)_ = 3.27, *p* = 0.0810]. No interaction was found.

## Discussion

This study shows that 45 day treatment with GUO reversed the hyperlocomotion and attenuated the memory deficit induced by OBX. The biochemical analysis of the hippocampi from those animals shows that treatment with GUO completely reversed the BDNF increase and the redox imbalance, as well as discreetly attenuated the pro-inflammatory status induced by OBX. Additionally, MicroPET imaging analysis indicates that GUO reduced the OBX-induced hippocampal increase in FDG metabolism. The behavioral and neurochemical effects promoted by chronic GUO treatment on the OBX model are similar to those reported for antidepressant agents used clinically (9, 14, 46).

Regarding the antidepressant behavioral potential of GUO, we previously showed that an acute single administration of GUO was already capable to reverse the anhedonic-like behavior, as well as the short-term recognition memory impairment observed after 15 days post OBX surgery (25). Noteworthy, only when chronically administered, as observed in the present study, GUO reversed the hyperlocomotion induced by OBX, whereas acute GUO, as ketamine, administration was ineffective (25). The effects are in line with our proposal that OBX model of depression depicts a time dependent development of depressive-like behaviors (15).

In contrast with the recognition deficit observed in shorter post-surgery periods (25), we show in the present study that 8 weeks after OBX surgery the recognition memory evaluated by NORT is partially recovered. As previously demonstrated for the transient loss of self-care and motivational behavior in OBX mice (15), the same pattern was here verified for recognition memory as assessed by NORT. Nevertheless, the lower discrimination rate of OBX mice at NORT suggests incomplete remission of long-term memory deficits within 2 months of surgery. It has been reported that GUO can promote amnesic effects (25, 47, 48). Amnesic effects were observed when GUO was administered acutely (25) or for a short time (up to 2 weeks) (48), but not after along 6 weeks treatment (49). In the current study, GUO did not induce any memory disturbance; on the contrary, GUO treatment for 8 weeks attenuated the residual memory deficit observed in untreated OBX mice (50). In agreement to our findings, it was recently reported that GUO administered during 26 days induces antidepressant-like effect, with no impairment on learning and memory (51). Taken together, those results suggest that a short-term disturbance in GUO levels can negatively impact learning and memory, but homeostasis is regained after repeated administration cancelling GUO amnesic effect.

In the present study, IMI treatment did not reverse the memory deficit induced by OBX in the YMT task. Similar results were reported for tricyclic antidepressants (52, 53), as well as for chronic IMI treatment on spatial working memory deficits in OBX mice tested 2 weeks after OBX (54). Similar results were found with rats treated with IMI for 10 or 28 days, resulting in impaired delayed in spatial win-shift performance (41). The results suggest that, differently than GUO, treatment with IMI presents a time independent impairment on learning and memory. The discrepancy may be related to the fact that as an endogenous compound GUO metabolism can be adjusted in accordance to GUO level.

Neurogenic and proliferative abilities are recognized for olfactory bulbs (43), with studies showing that after different types of lesions spontaneous recovery occurs over time (42, 44, 45, 55). To test the hypothesis that odor discrimination could be regained over time, we investigated the effects of time and treatment on Sham and OBX mice capability to discriminate odor. By using a modified NORT protocol (mNORT) where objects are smeared with rat odor, it was shown that OBX-induced anosmia was still present at follow-up. As in NORT, chronic GUO improved the OBX-induced residual memory deficit in mNORT. Therefore, the improvement or attenuation of memory impairment induced by OBX was the result of GUO treatment and not a potential recovery of odor discrimination.

Several neuroprotective effects of GUO have been demonstrated, but its exact mechanism of action is still unclear (17). Nevertheless, *in vivo* and *in vitro* studies evidenced that GUO modulates a broad range of cellular pathways that are closely related to various brain functions (16, 17). Notable, evidences support that MDD physiopathology is also related to alterations in some of those signaling and metabolic pathways (53). Besides neurotransmitter imbalance, altered levels of neurotrophic factors/neurogenesis, altered glial/neuronal biology, impairment in mitochondrial functionality, hypermetabolism in brain specific regions including hippocampus, increased proinflammatory scenario, and nitrosative and oxidative stress are also observed in the patients suffering from MDD, as well as in animals submitted to different models that mimic MDD (12, 56). Therefore, in the present study, we explored some of the neurochemical systems related to depression and previously observed to be modulated by GUO.

BDNF profiles were largely explored in several rodent models of MDD. The literature reveals a tight association between depression phenotype and decreased BDNF content, as well as its reversal by antidepressant when investigated in different stress-induced rodent models of depression (57). However, in the OBX model of depression, the literature suggests distinct hippocampal BDNF modulations (14). Accordingly, an age dependent opposite effect on BDNF level was observed after OBX surgery, as 10 week old mice submitted to OBX presented an up-regulation in BDNF levels (58), while a decrease was observed in older animals (6 months old) (57). Literature supports that BDNF levels decline during normal brain aging, which are often accompanied by mild brain atrophy, reduced neuronal function, and synaptic loss (59). Our result reinforces the data on young mice, as we also observed here an increase on BDNF levels in mice submitted to OBX at 7 weeks old. Considering the critical role of BDNF in synaptic plasticity (60, 61), the increment in BDNF content observed in the current study can be associated with the attenuation of behavioral deficits (NORT and YMT) in untreated OBX mice over time. Given that our data show that GUO prevented the OBX-induced BDNF increment and behavioral deficits, it is conceivable that the neuroprotective effect of GUO diminishes the need to increase BDNF. Alternatively, GUO could have an earlier effect on BDNF levels as it has been shown that GUO stimulates BDNF synthesis or its release inducing neuroplasticity in *in vitro* and *in vivo* experiments (27, 51, 62–66).

Chronic GUO completely reversed the OBX-induced hippocampal redox imbalance. The results corroborate previous data (67–70) indicating that GUO exerts its antioxidant action as a direct radical scavenger, preventing the OBX-induced increase in cellular ROS production, NO levels, and decrease in GSH content. The links among redox imbalance, inflammatory status, and depression are well-documented (71), including in our previous study exploring neurochemical changes in OBX mice model of depression, where we had demonstrated that a hippocampal pro-oxidative status is observed at 2 weeks after OBX, remaining dysregulated at least for more 6 weeks (15). Chronic GUO discreetly attenuated the pro-inflammatory status induced by OBX in mice hippocampi. Chronic GUO did not replicate the inhibitory effect on TNF-α release observed with acute GUO administration (69), or the anti-inflammatory effect obtained by subthreshold doses of GUO plus ketamine in mice submitted to a corticosterone model of depression (72). Overall, results indicate that the potential anti-inflammatory GUO property does not play a significant for the antidepressant effects of chronic GUO.

Numerous evidences pointed to a relevant role for the glutamatergic system in the pathophysiology of depression disorder (73, 74). Accordingly, it has been documented that different brain regions of OBX rodents are more sensitive to release glutamate when exposed to novelty (75–77). A limitation for this study is the absence of data on CSF glutamate content. Nevertheless, we show that OBX induced an increase in hippocampal glutamate uptake, unaffected by chronic GUO. Regarding the main astrocytic glutamate transporters, OBX mice presented decreased GLAST mRNA expression, with no changes in GLT-1, a pattern unchanged by chronic GUO. Considering that OBX increases glutamate release (75–77), it is arguable that increased glutamate uptake is to be expected. In fact, GUO stimulates glutamate uptake *in vitro* and *in vivo* (16, 17), but only in the presence of high glutamate concentration or neurological conditions, respectively (37). As OBX increases glutamate uptake for a long period, it is of interest to investigate, at different brain areas and time points, if GUO minimizes earlier glutamatergic excitotoxicity by increasing glutamate uptake and/or its use as energy substrate during recovery.

Alongside with the increased hippocampal glutamate uptake, OBX also induced increase in hippocampal FDG metabolism. The increase in FDG metabolism is present at baseline (15 days after surgery) and magnified at follow-up (60 days after surgery). The effect is more prominent in the hippocampus dorsal region in comparison to the ventral part. Although previous studies suggested that glucose hypermetabolism might result from increased gliosis (78), we did not identify changes in GFAP astrocytic marker. Further investigation with a broader range of glial markers is necessary to clarify this effect. Stimulation of the glycolytic pathway (mainly in astrocytes) has been associated to increased BDNF signaling (79), increased levels of oxidative and nitrosative radicals (80), enhancement in inflammatory cytokines (81), and/or increment in glutamate release (82). It is therefore arguable that the increment in [^18^F]FDG signal (83, 84) observed in untreated OBX mice may result from alterations in multiple brain signaling pathways. The increased in glutamate uptake accompained by increased glucose metabolism and BDNF levels is suggestive of a long-lasting plasticity process in the hippocampus of mice subjected to OBX. Forty-five days of GUO treatment prevented the OBX-induced increase in hippocampal FDG metabolism in untreated animals. The lack of increase in FDG metabolism accompanied by attenuated BDNF increase and diminished redox imbalance corroborate the hypothesis that GUO modulates different brain pathways contributing, directly or indirectly, to a better-balanced brain metabolism.

## Conclusion

Our data clearly show that chronic GUO attenuates the behavioral changes induced by OBX, an effect accompanied by neurochemical changes associated with hippocampal plasticity. The antidepressant effect elicited by GUO seems to be associated with its ability to concurrently prevent OBX-induced BDNF increment and redox imbalance, as well as increase in FDG metabolism.

Adding to previous reports showing that acute GUO acts as a fast-onset antidepressant (25), the present data suggest continued benefits with chronic treatment. This result is of value since data suggest that GUO systemic administration is safe, well-tolerated, and not associated with major side effects (85, 86). Additionally, our data reinforces the role of the purinergic system in MDD physiopathology.

## Data Availability Statement

The original contributions presented in the study are included in the article/Supplementary Material, further inquiries can be directed to the corresponding author/s.

## Ethics Statement

The animal study was reviewed and approved by Comissão de Ética no Uso de Animais (CEUA/UFRGS) project number #24577.

## Author Contributions

RFA was responsible for the design, acquisition, analysis, interpretation, drafting, and approval of the final version of the manuscript. YN, SL, AR, DGM, BB, FF, DL, LP, GV, and SG were responsible for the acquisition of some data displayed in the manuscript. JC was responsible for the *in vivo* microPET scans. EE, MG, EZ, and DS were responsible for the interpretation, drafting, critical revision, and approval of the final version of the manuscript.

## Conflict of Interest

The authors declare that the research was conducted in the absence of any commercial or financial relationships that could be construed as a potential conflict of interest.

## Publisher's Note

All claims expressed in this article are solely those of the authors and do not necessarily represent those of their affiliated organizations, or those of the publisher, the editors and the reviewers. Any product that may be evaluated in this article, or claim that may be made by its manufacturer, is not guaranteed or endorsed by the publisher.

## Supplementary Material

Supplementary Figure 1 Representative image of the lateral septal nucleus VOI template, used as a reference region for the SUVr normalization **(A)**. Representative images of the total hippocampus **(B)** and subregions, dorsal **(C)** and ventral **(D)** VOI templates. Representative images of the hippocampal positive (metabolism increase) percentage of change between group Sham Sal baseline and groups Sham GUO, OBX Sal, and OBX GUO, in the baseline scan **(E)**. Representative images of the hippocampal positive (metabolism increase) percentage of change between group Sham Sal baseline and groups Sham Sal, Sham GUO, OBX Sal, and OBX GUO, in the follow-up scan **(F)**. T-statistical maps showing the statistically significant increased metabolism in the hippocampal region on groups Sham GUO, OBX Sal, and OBX GUO, in the follow-up scan, in comparison with the group Sham Sal follow-up **(G)**. Representative images of the hippocampal positive (metabolism increase) percentage of change between group Sham Sal follow-up and groups Sham Sal, Sham GUO, OBX Sal, and OBX GUO, in the follow-up scan **(H)**.
