# Supplementary figures and images for: Antidepressant-Like Effects of Chronic Guanosine in the Olfactory Bulbectomy Mouse Model
Source: Front Psychiatry. 2021 Aug 4;12:701408. doi: 10.3389/fpsyt.2021.701408 (PMC8371253; doi:10.3389/fpsyt.2021.701408)

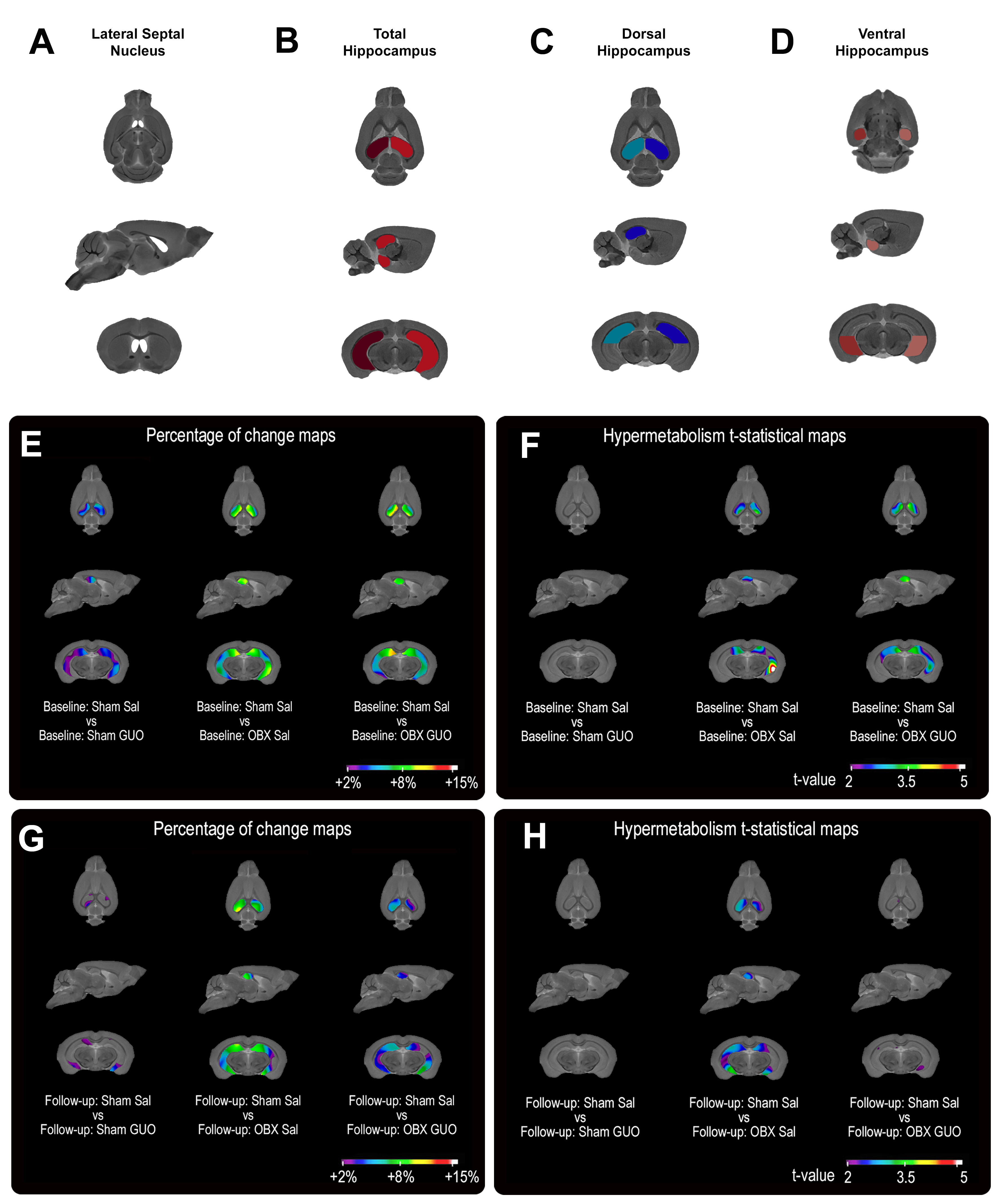

Supplement: Supplementary Figure 1 — Representative image of the lateral septal nucleus VOI template, used as a reference region for the SUVr normalization (A). Representative images of the total hippocampus (B) and subregions, dorsal (C) and ventral (D) VOI templates. Representative images of the hippocampal positive (metabolism increase) percentage of change between group Sham Sal baseline and groups Sham GUO, OBX Sal, and OBX GUO, in the baseline scan (E). Representative images of the hippocampal positive (metabolism increase) percentage of change between group Sham Sal baseline and groups Sham Sal, Sham GUO, OBX Sal, and OBX GUO, in the follow-up scan (F). T-statistical maps showing the statistically significant increased metabolism in the hippocampal region on groups Sham GUO, OBX Sal, and OBX GUO, in the follow-up scan, in comparison with the group Sham Sal follow-up (G). Representative images of the hippocampal positive (metabolism increase) percentage of change between group Sham Sal follow-up and groups Sham Sal, Sham GUO, OBX Sal, and OBX GUO, in the follow-up scan (H). [file Image_1.TIFF]
